# Soil recovery across a chronosequence of restored wetlands in the Florida Everglades

**DOI:** 10.1038/srep17630

**Published:** 2015-12-01

**Authors:** Qibing Wang, Yuncong Li, Min Zhang

**Affiliations:** 1 State Key Laboratory of Vegetation and Environmental Change, Institute of Botany, the Chinese Academy of Sciences, 20 Nanxincun Xiangshan, Beijing 100093, China; 2 Department of Soil and Water Science, Tropical Research and Education Center, University of Florida, Homestead, FL 33031, USA; 3 College of Resources and Environment, Shandong Agricultural University, Taian, Shandong Province 271018, China

## Abstract

The restoration project in the Hole-in-the-Donut of Everglades National Park in Florida, USA is to reestablish native wetlands by complete removal of the invasive plants and the associated soil. However, there is little information available about changes in properties of the newly formed Marl soils in restored wetlands. In this study, we measured soil physicochemical properties, soil enzymatic activities, and stable isotopes of carbon (δ^13^C) in plants and soil organic carbon (SOC) in an undisturbed natural wetland (UNW) and three wetlands restored respectively in 1989, 1996 and 1999 (WR89, WR96 and WR99). The older restored wetlands (WR89 and WR96) are characterized by greater SOC and mineral nitrogen. The values of soil dehydrogenase and phosphatase activities in the four wetlands follow the order: UNW > WR89 > WR96 > WR99, and are consistent with changes in vegetation coverage. The principal component analysis shows that dehydrogenase and phosphatase activities are the vital variables contributing to the soil of UNW. The similar δ^13^C values of SOC and plants in the restored wetlands suggest the formation of SOC during restoration is mainly derived from the associated plants. These results indicate that the newly restored soils develop toward the soil in the UNW with time since restoration.

Wetlands have long been the spotlight in biodiversity, although they only cover 6% of the total world surface. Influenced by environmental stress and human activities, the world’s wetlands are facing threats. About 50% of wetlands in the world have disappeared since 1900. Tropical and subtropical wetlands have been the main victims since the 1950s^1^. Therefore, the restoration of wetlands calls for immediate attention.

Scientific studies surrounding the effects of wetland restoration on ecosystem functioning are pivotal and would have useful management implications. Such studies have been mostly focused on the restoration of native plant communities^2^,^3^. However, there are few studies addressing how the soil changes in response to wetland restoration. Soil forms an integral part and plays an important role in shaping plant species composition and vegetation structure of an ecosystem^4^. Therefore, it is vital to assess soil quality of restored wetlands in the development of sustainable wetland ecosystems. Soil quality depends on physical, chemical, biochemical and biological properties of the soil^5^, especially the soil properties (e.g. the activities of hydrolytic enzymes) that are sensitive to anthropogenic disturbances^6^. Soil enzyme activities are frequently measured to evaluate ecosystem function^7^,^8^. For the success in wetland restoration practice, a comprehensive understanding of the quality of the newly restored soil is needed.

Everglades National Park in Florida is the largest subtropical wilderness in the United States, and covers the southern 25% of the original Everglades marshland region of southwestern Florida. As a fragile ecosystem, a large area of Everglades National Park has been invaded by exotic pest plants, Brazilian pepper (*Schinus terebinthifolius* Raddi), because of high disturbance by previous farming^9^. A restoration scheme in which invasive plants and all the associated soil are completely removed down to the limestone bedrock has been ongoing in the park since 1989^9^. It is necessary to evaluate the changes in the soil properties of restored wetlands and to establish the evidence for predicting the effectiveness of wetland restoration in the longterm. The overall objectives of our study were (1) to document the changes in the physical, chemical and biochemical properties of the soil along a restoration chronosequence and (2) to evaluate soil recovery with time since restoration by comparing the soil properties in three restored wetlands with those in an adjacent undisturbed native wetland.

## Results

The soil depth, moisture and soil water holding capacity in four sites (i.e., an undisturbed natural wetland (UNW) and three wetlands restored respectively in 1989, 1996 and 1999 (WR89, WR96 and WR99)) followed the order: UNW > WR89 > WR96 > WR99 (Table 1). Soil texture measurements show that the restored wetland soils are loamy fine sands and fine sandy loams with 14–16% clay and 23–36% sand, both of which are significantly greater than those in UNW, and the soil in the undisturbed natural wetland has significantly more silt than all the restored wetland soils (82% silt in UNW).

The soil pH values ranged from 7.6 to 7.7 and there were no significant differences among UNW, WR89, WR96 and WR99 (Table 2). The greatest soil electric conductivity (EC) was 0.46 dS/m in WR89 and WR96, whereas the smallest was 0.32 dS/m in UNW. The soil organic carbon (SOC) concentration in WR99 was the smallest among the four sites. The soil total N concentration in UNW was greater than that in WR89 or WR99. The C/N ratios were consistent among the four sites. The correlation analysis showed that the total N concentrations were highly correlated to the SOC concentrations (*r* = 0.87, *P* < 0.01). The mineral N (NH_4_-N and NO_3_-N) concentrations in the soils of WR89 and WR96 were significantly greater than those in the soils of UNW and WR99. The newly formed soils had similar calcium carbonate concentrations to the natural wetland soil. The mean total P and Cu concentrations in all the soils of the restored wetlands were significantly greater than those in the soil of UNW. Statistically, there were no significant differences in the concentrations of K, Ca, Mg, Fe, Mn and Zn among the soils of the four sites, except for the Mn concentration in the soil of WR89.

The δ^13^C values in plant tissues were not significantly different in the four wetlands, ranging from −24.6 to −25.9% (Table 3). The δ^13^C values of SOC in the restored wetlands decreased with the increase in restoration duration (−23.7, −24.7 and −26.5% in WR99, WR96 and WR89, respectively), and the greatest δ^13^C value of SOC was found in UNW (−21.9%).

**Figure 1:**
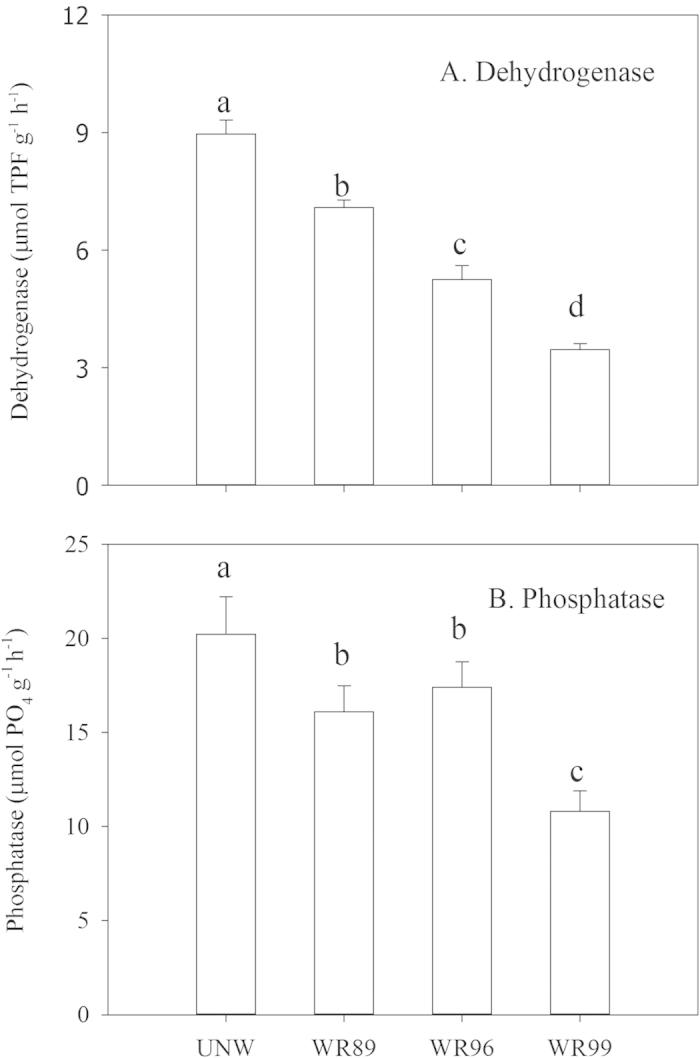
Dehydrogenase and phosphatase activities in the soils of an undisturbed natural wetland (UNW) and the wetlands restored respectively in 1989, 1996 and 1999 (WR89, WR96 and WR99) in the Hole-in-the-Donut (HID), Everglades National Park. Vertical bars are standard errors. Means followed by the same letters are not significantly different by Duncan’s multiple range test at *P* < 0.05, n = 5.

Both of the dehydrogenase and phosphatase activities in the soil of UNW were significantly greater than those in the soils of the restored wetlands (Fig. 1). Overall, these enzyme activities were significantly enhanced with time since restoration.

**Figure 2:**
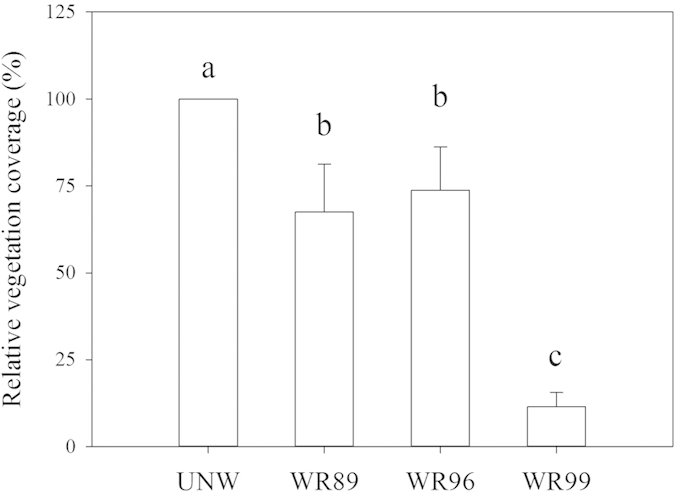
Relative **v**egetation coverages in an undisturbed natural wetland (UNW) and the wetlands restored respectively in 1989, 1996 and 1999 (WR89, WR96 and WR99) in the Hole-in-the-Donut (HID), Everglades National Park. Vertical bars are standard errors. Means followed by the same letters are not significantly different by Duncan’s multiple range test at *P* < 0.05, n = 5.

Figure 2 presents the mean values of vegetation coverage in WR99, WR96 and WR89 relative to UNW. The total coverage increased significantly from 12% in WR99 to 74 and 68% respectively in WR96 and WR89.

**Figure 3:**
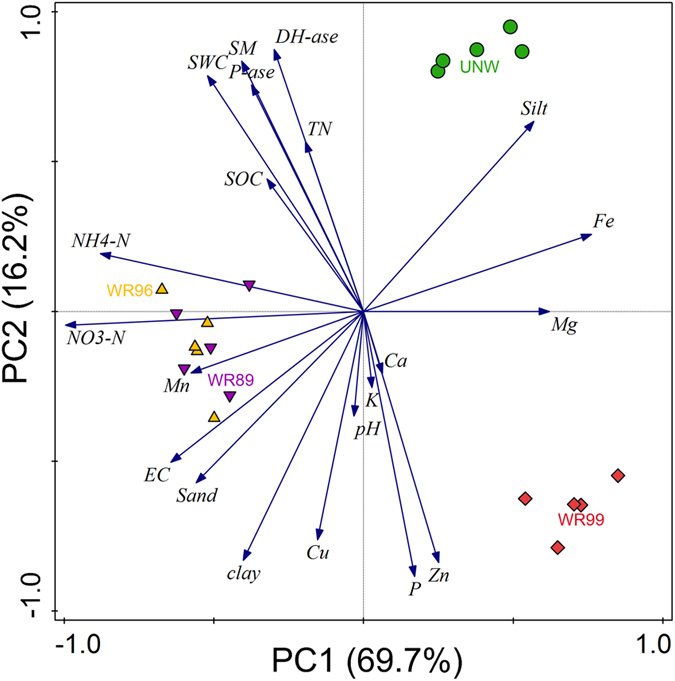
A biplot of principal component analysis (PC1 and PC2) of soil properties in an undisturbed natural wetland and the wetlands restored respectively in 1989, 1996 and 1999 (WR89, WR96 and WR99) in the Hole-in-the-Donut (HID), Everglades National Park. SM, soil moisture; SWC, soil water holding capacity; EC, electric conductivity; SOC, soil organic carbon; TN, total N; DH-ase, dehydrogenase activities; P-ase, phosphatase activities.

Using principal component analysis (PCA), we are able to summarize the similarity and dissimilarity in soil properties in relation to the undisturbed natural wetland and the wetlands across the restoration chronosequence. The first two principle components (PC1 and PC2) explain 85.9% of the variation in the soil properties, with PC1 accounting for 69.7% and PC2 accounting for 16.2% (Fig. 3), showing that most information about soil properties was contained in the first two principle components. Biochemical variables, i.e., dehydrogenase and phosphatase activities, were positively correlated with soil moisture, water holding capacity, total N and SOC (Fig. 3). The distance between the different wetlands in the biplot of PCA shows that the soil properties of the older restored wetlands WR89 and WR96 were similar. Compared with the cluster WR99, the cluster of WR89 and WR96 is closer to the cluster UNW (Fig. 3).

## Discussion

The depth of Perrine Marl soil ranges from 3–34 cm, with a typical depth of 11 cm, i.e., the soil depth of UNW. We found that averagely the soil depth increased by 0.2–0.3 cm per year during wetland restoration. Scholl, *et al*. used radiocarbon dating to estimate the deposition rate of the Marl soil in present coastal areas, and found that the average rate was 1.2 cm/100 years over one thousand years^10^. The greater soil formation rate compared with the historical average rate needs a further investigation. The evacuation of plants and soil on the limestone bedrock lowered elevation in the wetlands of exotic plant invasion^2^; thus the lower elevation increased the inflow of the surface water outside the restoration area, and carried in significant quantities of periphyton that attaches to limestone and soil particles, which would facilitate the soil formation. Soil water holding capacity is usually considered to be one of the most important factors that impact wetland plant community composition, because wetland plants are adapted to specific hydrological regimes^11^. Therefore, successful wetland reconstruction must first restore the soil water holding capacity of a wetland^12^. In our study, the soil water holding capacity in the restored wetlands presented an increasing trend with restoration time, approaching to that in the undisturbed natural wetland.

The soil pH values in the four sites are typical for the calcareous soils in South Florida. The consistent pH among the four wetlands suggests that the soil pH may resist to heavy disturbance in this area. The soil EC in our study was consistent with the finding of Li & Norland^13^ that the soil EC in the undisturbed natural wetland was smaller than that in the restored wetland, which could be attributed to the previous fertilizer application during the agricultural production. As a key element of soil property, SOC represents an informative index of wetland restoration^14^,^15^. Our principal component analysis suggests that SOC has the similar contribution to the soils of WR89 and WR96 (Fig. 3). Tisdale *et al*. reported that soils with C/N below 20 could offer sufficient N for plant uptake^16^; thus our result on the soil C/N (13.5–15.7) of the three restored wetlands suggests that all the newly formed soils can provide adequate N for the associated native plants to be recovered. Chen *et al*. reported that the mean total P was 0.22 g/kg in the undisturbed Marl soil in South Everglades^17^. In the present study, the mean total P in the soil of UNW was 0.16 g/kg. Total P and Cu concentrations in the soils of the restored wetlands were significantly greater than those in the soil of the undisturbed natural wetland in our study. Li & Norland reported the similar results, and found that soil P and Cu concentrations in the restored wetland and the rock-plowed farmland were statistically same, while they were roughly 4 times those in the undisturbed natural wetland^13^. It is very likely that the frequent raining in the study areas results in P and Cu leaching into the interface of soil and bedrock. Thus, the soils in the restored wetlands retained relatively great amounts of P and Cu.

In the same sites, a detail investigation on the species composition of vegetation carried out by the Everglades Research Group showed that 61–73% of the plant species in the restored wetlands were natural wetland associated species of that region^18^. Their finding supports our results that δ^13^C values of plants were similar among the undisturbed natural wetland and the restored wetlands. These results make a strong case to prove that the native wetland vegetative communities were successfully restored after the complete removal of non-native plants and the associated soil in Everglades National Park. In the three restored wetlands, the δ^13^C values of SOC closed to that of plants. It suggests that the formation of soil organic carbon during the wetland restoration is mainly affected by the plants.

Soil enzyme activity is an integral index of soil quality^19^. Soil hydrolase, which includes dehydrogenase and phosphatase, is one of the most important soil enzymes in evaluating soil quality^4^. Dehydrogenase activity can reflect the oxidative capacity of soil microorganisms^20^,^21^. Inglett *et al*. examined the soil microbial community composition using phospholipid fatty acid analysis (PLFA) across the restored and the undisturbed natural wetlands in the similar study sites^22^. They observed that microbial biomass C and total PLFA increased with time since restoration and gradually approached to those in the undisturbed natural wetland^22^, which is in line with the trends of the dehydrogenase and phosphatase activities in our study. Enzyme activities indicate the potential of the soil biochemical processes, which is essential for the maintenance of soil quality^23^. The principal component analysis indicates that dehydrogenase and phosphatase activities are the vital contributors to the soil of the undisturbed natural wetland (Fig. 3). And, the soil dehydrogenase and phosphatase activities increased with time since restoration and gradually approached to those in the undisturbed natural wetland (Fig. 1). Therefore, these results suggest that the newly restored soils develop toward the soil in the undisturbed natural wetland with time since restoration.

The development of the newly formed soils in the restored wetlands laid the foundation for return of native Marl wetland vegetation. In general, the changes in the vegetation coverage were consistent with those in the soil enzyme activities in the four wetlands.

In summary, the newly restored Marl soils and the associated native plants were mutually reinforcing and both were recovered to some extent toward the native wetland along the short-term restoration chronosequence. It suggests the success in the wetland restoration approach of complete removal of the plants as well as the associated soil. The results from the present study provide critical scientific information for evaluation and management of wetland ecosystem restoration practices worldwide in the long term.

## Materials and Methods

### Study area

The study was carried out in the Hole-in-the-Donut (HID), which is located in the eastern half of Everglades National Park, Florida, USA (N25^o^22′32′′, W80^o^37′27′′). The Everglades has a sub-tropical climate with a mild winter from December to April (average monthly temperature ranges from 12 to 25 °C) and a hot and humid summer (average monthly temperature is approximately 32 °C). The mean annual rainfall is 1520 mm and the rainy season is June through October. The natural wetland soil in the southeastern Everglades is Marl, which is classified as Perrine Marl (Coarse-silty, carbonatic, hyperthermic Typic Fluvaquents) in the soil survey^24^.

The HID, which covers an area of approximately 4000 ha, is a major site of exotic plant invasion. In particular, Brazilian pepper (*Schinus terebinthifolius* Raddi), which is the most noxious invader in the area, created a canopy so thick that native vegetation could not grow in its understory. This area was originally composed of sawgrass (*Cladium jamaicense* Crantz) prairie, pineland and some hammocks^2^,^13^,^25^. The exotic plant invasion is related to rock-plowing of the substrate and intensive vegetable crop production in the area^26^. The rock–plowing that was introduced in the early 1950s increased the soil depth by crushing natural oolitic limestone bedrock into a growth medium, and thus significantly improved vegetable crop production. However, the soil under the vegetable crop production received high levels of chemical fertilizers and pesticides^26^,^27^, which consequently altered the physical and chemical properties of the soil and left the area susceptible to intensive invasion of exotic plants^13^. The farming operations were discontinued in 1975; thereafter, the HID was left abandoned and soon became a mono-specific stand of the non-native plant Brazilian pepper^28^.

In order to restore the HID area to a Marl wetland vegetative community with its associated wildlife, various *S. terebinthifolius* control methods, such as soil compacting, planting natives, mowing, burning, bulldozing, etc., have ever been applied to suppress this invader in the HID, but none has been efficient^3^,^29^,^30^. In 1989, a new experiment was carried out. In this experiment, the rock-plowed soil was scraped down to the consolidated limestone, and the soil and the associated plants were completely removed from an experimental area of 18.2 ha. Two to seven months after the removal, the native plants were reestablished on the restored wetland^2^. Everglades National Park initiated a restoration project with this soil removal approach to eliminate Brazilian pepper and restore the native wetland vegetation in the whole area in 1996^31^. By July 2001, about 18% of the HID had been restored^9^.

### Field sampling

We chose four sampling sites in the HID: 1) undisturbed natural wetland; 2) wetlands restored in 1989; 3) wetlands restored in 1996; 4) wetlands restored in 1999. The total coverage of vegetation was estimated using a line intercept method following McDonald, Isbell, Speight, Walker and Hopkins^32^. In each site, we randomly selected 5 sampling plots (1 m × 1 m) as replicates. In each plot, soil depth was measured, and a 1-kg soil sample was collected from surface to bedrock and mixed thoroughly. All samples were stored in sealed polyethylene bags, and transported to the laboratory within 3 hours. About 100 g of each soil sample was kept at 4 °C in a refrigerator for soil enzyme activity analysis. The rest of the soil samples were air-dried at room temperature, ground to pass through a 2-mm stainless steel screen, and stored in lined paper bags until analysis. A soil subsample of about 100g was for the analysis of soil physical and chemical properties. Another soil subsample of about 100 g was dried at 60 °C, and ground to pass through a 1-mm sieve for the analysis of stable carbon isotope (δ^13^C).

Plants in each plot were cut at ground level and placed into polyethylene bags. These samples were washed with deionized water, and dried at 70 °C in an oven. Approximately 50 g of each dry sample was ground by a stainless steel mill, and stored in a sealed plastic bag for δ^13^C analysis.

### Laboratory analyses

Soil moisture was measured by gravimetric method. Soil water holding capacity was determined by measuring the gravimetric water content of the sieved soil that was saturated in deionized water and allowed to drain for 24 hours in a filter funnel at 100% humidity^33^,^34^. Soil particle distribution was determined by a micropipette method^35^. Soil pH and EC were measured by Multilab P4 (Multiparameter Instrument) (Wissenschaftlich-Technische Werkstatten GmbH, Weilheim, Germany) in water (soil/water ratio of 1:2). Soil organic carbon was determined by weight loss-on-ignition method at 450 °C for 24 hours^35^. Total N was analyzed through the Kjeldahl digestion method^28^. Soil NH_4_-N and NO_3_-N were extracted with 2 M KCl, and analyzed by an AutoAnalyzer III (Bran and Luebbe, Hamburg, Germany). Soil carbonate concentration was determined by tritration^36^. Soil samples were digested according to EPA method 3050A^37^, and analyzed for total concentrations of P, K, Ca, Mg, Fe, Mn, Cu and Zn using an ICP/OES Optima 3000 inductively coupled plasma emission spectrometer (PerkinElmer, Waltham, Massachusetts, USA).

The carbon isotope ratios in SOC and in plant were both determined by analyzing the CO_2_ resulting from the combustion of a sample together with CuO and silver foil in evacuated quartz tubing at 900 °C. The CO_2_ generated by the combustion was cryogenically extracted and its stable C isotopic ratios were measured on a Finnigan MAT Delta S isotope ratio mass spectrometer (Thermo Fisher Scientific Inc., Waltham, Massachusetts, USA)^38^. The analytical precision is 0.1%. The carbon stable isotope data are reported relative to PDB standard (a Cretaceous mollusk) as:


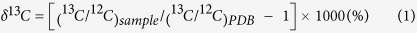


Soil dehydrogenase activity was determined by the reduction of 2,3,5-triphenyltetrazolium chloride (TTC) to triphenyl formazan (TPF) by soil microorganisms^39^,^40^. Briefly, the mixture of 1 g soil sample with 0.6 ml 3% aqueous solution of TTC was incubated at 37 °C for 24 h, extracted with 10 ml methanol, and centrifuged at 12000 rpm for 10 min. The concentration of the TPF in the supernatant was measured spectrophotometrically at the 485 nm wavelength.

Soil phosphatase activity was determined as the amount of orthophosphate released in the soil incubated with buffered (pH 8.0) pyrophosphate solution^41^. Briefly, the mixture of 1g soil sample with 3 ml of 50 mM pyrophosphate solution was incubated at 37 °C for 5 h. The orthophosphate released during the incubation was extracted with H_2_SO_4_ in a modified universal buffer solution, and determined immediately by a spectrophotometer at 700 nm.

### Data analysis

Relative vegetation coverage is calculated as the coverage of a given restored wetland divided by that of the undisturbed natural wetland. One-way analysis of variance was performed with the General Linear Model (SAS 8.0) procedure to test significance for the soil property parameters across the study sites^42^. Means were separated by Duncan’s multiple range test at 5 and 1% levels. Correlation coefficients among variables were also calculated by the SAS software. Principal component analysis (PCA) was performed to characterize the similarity in soil properties among the four wetlands using Canoco 5.0 (Biometris-Plant Research International, Wageningen).

## Additional Information

**How to cite this article**: Wang, Q. *et al*. Soil recovery across a chronosequence of restored wetlands in the Florida Everglades. *Sci. Rep.* **5**, 17630; doi: 10.1038/srep17630 (2015).

## Figures and Tables

**Table 1:** Soil physical properties in an undisturbed natural wetland (UNW) and the wetlands restored respectively in 1989, 1996 and 1999 (WR89, WR96 and WR99) in the Hole-in-the-Donut (HID), Everglades National Park.

Variable	UNW	WR89	WR96	WR99
Depth (cm)	11.1 (2.6)a	4.6(0.6)ab	3.0 (0.8)bc	2.1(0.4)c
Moisture (%)	75.1(6.0)a	61.7(3.9)ab	51.5(3.2)b	25.5(2.2)c
SWC* (%)	120.1(2.4)a	112.7(5.3)a	109.0(4.7)a	77.0(3.9)b
Clay (%)	8.4(0.1)b	14.1(0.4)a	15.7(0.6)a	14.8(1.1)a
Silt (%)	82.3(1.4)a	49.9(2.6)c	56.6(5.0)c	62.4(5.0)b
Sand (%)	9.3(1.3)c	36.1(3.0)a	27.8(5.4)ab	22.8(4.2)b

Values are means with standard errors in parentheses. Means in the same row followed by the same letters are not significantly different by Duncan’s multiple range test at *P* < 0.05, n = 5.

^*^SWC, soil water holding capacity.

**Table 2:** Soil chemical properties in an undisturbed natural wetland (UNW) and the wetlands restored respectively in 1989, 1996 and 1999 (WR89, WR96 and WR99) in the Hole-in-the-Donut (HID), Everglades National Park.

Variable	UNW	WR89	WR96	WR99
pH	7.64 (0.06)a	7.70 (0.02)a	7.66 (0.04)a	7.71 (0.03)a
EC* (dS m^−1^)	0.32 (0.23)b	0.46 (0.19)a	0.46 (0.23)a	0.39 (0.14)ab
SOC (mg g^−1^)	71.53 (4.65)a	58.80 (4.76)b	80.18 (7.03)a	50.43 (5.77)b
Total N (mg g^−1^)	5.99 (0.78)a	3.94 (0.49)b	5.93 (0.59)a	3.76 (0.33)b
C/N Ratio	12.71 (2.12)a	15.70 (3.44)a	13.56 (0.38)a	13.53 (1.42)a
NH_4_ (μg g^−1^)	12.63 (0.52)a	16.88 (1.20)a	18.00 (1.22)a	9.88 (0.55)a
NO_3_ (μg g^−1^)	6.25 (1.44)b	48.50 (7.01)a	48.50 (3.04)a	3.25 (0.66)b
Mineral N (μg g^−1^)	18.88 (0.94)b	65.67 (6.71)a	67.50 (3.91)a	13.13 (0.24)b
CaCO_3_ (%)	78.66 (1.17)a	82.46 (2.85)a	80.67 (2.66)a	82.29 (1.39)a
P (mg g^−1^)	0.164 (0.01)c	0.355 (0.06)b	0.407 (0.08)b	0.601 (0.06)a
K (mg g^−1^)	0.414 (0.05)a	0.461 (0.07)a	0.445 (0.10)a	0.446 (0.02)a
Ca (mg g^−1^)	295.6 (42.01)a	312.7 (16.34)a	298.3 (20.09)a	321.6 (8.78)a
Mg (mg g^−1^)	1.670 (0.20)a	1.283 (0.20)a	1.275 (0.24)a	1.685 (0.05)a
Fe (mg g^−1^)	12.16 (2.43)a	6.290(1.50)a	4.013 (0.62)a	9.643 (1.43)a
Mn (mg g^−1^)	0.146 (0.01)b	0.576 (0.12)a	0.268 (0.04)b	0.202 (0.02)b
Cu (μg g^−1^)	3.80 (0.50)b	4.93 (0.16)ab	5.37 (0.63)a	5.37(0.21)a
Zn (μg g^−1^)	7.62 (0.64)a	13.74 (3.15)a	11.05 (1.59 )a	20.28 (2.29)a

^*^Note: EC, electric conductivity.

Values are means with standard errors in parentheses. Means in the same row followed by the same letters are not significantly different by Duncan’s multiple range test at *P* < 0.05, n = 5.

**Table 3:** δ^13^C (%) in plant and soil from an undisturbed natural wetland (UNW) and the wetlands restored respectively in 1989, 1996 and 1999 (WR89, WR96 and WR99) in the Hole-in-the-Donut (HID), Everglades National Park.

Variable	UNW	WR89	WR96	WR99
Plant	−25.93 (0.12)a	−24.77(0.17)a	**−**24.60 (0.60)a	−24.97 (0.94)a
SOC	−21.90 (0.40)a	−26.53 (0.50)c	−24.73 (0.59)bc	−23.67 (1.10)ab

Values are means with standard errors in parentheses. Means followed by the same letters in the each column are not significantly different by Duncan’s multiple range test at *P* < 0.05, n = 5.
